# Surveillance in eastern India (2007-2009) revealed reassortment event involving ns and PB1-F2 gene segments among co-circulating influenza a subtypes

**DOI:** 10.1186/1743-422X-9-3

**Published:** 2012-01-05

**Authors:** Mehuli Sarkar, Shampa Chanda, Sekhar Chakrabarti, Jaydeep Mazumdar, Anirban Ganguly, Mandeep S Chadha, Akhilesh C Mishra, Mamta Chawla-Sarkar

**Affiliations:** 1 Division of Virology, National Institute of Cholera and Enteric Diseases, P-33, C.I.T. Road, Scheme XM, Beliaghata, Kolkata 700 010, West Bengal, India; 2 National Institute of Virology, 20A Ambedkar Road, Pune 411 001, India

**Keywords:** NS, PB1-F2, A/H1N1, A/H3N2, Reassortment

## Abstract

**Background:**

Influenza A virus encodes for eleven proteins, of which HA, NA, NS1 and PB1-F2 have been implicated in viral pathogenicity and virulence. Thus, in addition to the HA and NA gene segments, monitoring diversity of NS1 and PB1-F2 is also important.

**Methods:**

55 out of 166 circulating influenza A strains (31 H1N1 and 24 H3N2) were randomly picked during 2007-2009 and NS and PB1-F2 genes were sequenced. Phylogenetic analysis was carried out with reference to the prototype strains, concurrent vaccine strains and other reference strains isolated world wide.

**Results:**

Comparative analysis of both nucleotide and deduced amino acid sequences, revealed presence of NS gene with A/PR/8/34(H1N1)-like mutations (H4N, Q21R, A22V, K44R, N53D, C59R, V60A, F103S and M106I) in both RNA-binding and effector domain of NS1 protein, and G63E, the HPAI-H5N1-like mutation in NEP/NS2 of five A/H1N1 strains of 2007 and 2009. NS1 of other A/H1N1 strains clustered with concurrent A/H1N1 vaccine strains. Of 31 A/H1N1 strains, five had PB1-F2 similar to the H3N2 strains; six had non-functional PB1-F2 protein (11 amino acids) similar to the 2009 pandemic H1N1 strains and rest 20 strains had 57 amino acids PB1-F2 protein, similar to concurrent A/H1N1 vaccine strain. Interestingly, three A/H1N1 strains with H3N2-like PB1-F2 protein carried primitive PR8-like NS gene. Full gene sequencing of PB1 gene confirmed presence of H3N2-like PB1 gene in these A/H1N1 strains.

**Conclusion:**

Overall the study highlights reassortment event involving gene segments other than HA and NA in the co-circulating A/H1N1 and A/H3N2 strains and their importance in complexity of influenza virus genetics. In contrast, NS and PB1-F2 genes of all A/H3N2 eastern India strains were highly conserved and homologous to the concurrent A/H3N2 vaccine strains suggesting that these gene segments of H3N2 viruses are evolutionarily more stable compared to H1N1 viruses.

## Background

Influenza A virus (IAV) is a cytolytic virus that is responsible for significant morbidity and mortality worldwide per year. The genome of IAV consists of eight single-stranded, negative- sense viral RNA segments encoding the subunits of the transcriptase complex (PB1, PB2, PA), nucleoprotein (NP), the matrix protein (M1), two non-structural proteins (NS1 and NS2/NEP), three integral membrane proteins (hemagglutinin (HA), neuraminidase (NA) and proton channel (M2)) and the eleventh gene product PB1-F2 which is encoded by an alternative ORF of segment 2 [1]. Due to the segmented RNA genome, multiple subtypes, large number of hosts, IAVs cause yearly seasonal epidemics and have caused four pandemics in the last 100 years. Thus, there is an intense interest in understanding genomic diversity of virus encoded genes implicated in pathogenicity of diseases.

One such virulence factor is NS1, which is a multifunctional protein of IAV having role in suppression of host immune and apoptotic responses [2,3]. The major role of NS1 is to antagonize the antiviral response of the host by preventing the activation of NF-*κ*B and induction of alpha/beta interferon (IFN-α/β) [4]. It is additionally involved in (i) inhibiting the pre-mRNA 3'- end processing by binding to two 3'- end processing factors, namely cleavage and polyadenylation specificity factor and poly(A)- binding protein II [5-7]; (ii) blocking the post-transcriptional processing and nuclear export of cellular mRNA [6]; (iii) stimulating the translation of matrix (M1) proteins [8,9]; (iv) inhibiting the activation of a protein kinase that phosphorylates the eIF-2 translation initiation factor by binding to double stranded (ds) RNA [10,11], (v) induction of the phosphatidylinositol-3-kinase (PI3K/Akt) signaling pathway in order to support viral replication [12]. Additionally, a 15 kDA nuclear export protein (NEP, formally called NS2) translated from spliced mRNA of NS gene, mediates the export of viral ribonucleoproteins from the nucleus to the cytoplasm through nuclear export signals and is involved in independent interaction with human chromosome region maintenance protein Crm1 [13,14], as well as in viral assembly through its interaction with the M1 protein [15]. The second virulent factor PB1-F2 is encoded in the +1 reading frame of the PB1 gene and is translated from an AUG codon downstream of the PB1 start site, probably through a leaky ribosomal scanning [16]. It has been shown to contribute to virulence both directly and indirectly, through modulation of responses to bacteria [17,18].The exact mechanism(s) through which virulence is increased due to PB1-F2 expression is still not clear. Though based on overexpression studies, PB1-F2 has been shown to cause cell death in some cell types [1,19], induce inflammation by recruitment of inflammatory cells in mice [18] and to bind to PB1 resulting in increased activity of the influenza virus polymerase *in vitro *[20].

Since NS1 and PB1-F2 proteins have important role in viral pathogenicity, the aim of this study was a comprehensive evaluation of the IAV gene sequences encoding NS1 and PB1-F2 (segment 8 and segment 2) to understand evolution and genetic diversity of PB1-F2 and NS1 as well as NEP/NS2 in A/H1N1 and A/H3N2 strains circulating in eastern India during 2007-2009.

## Results

### Sequence analysis of the NS gene

**Figure 1:**
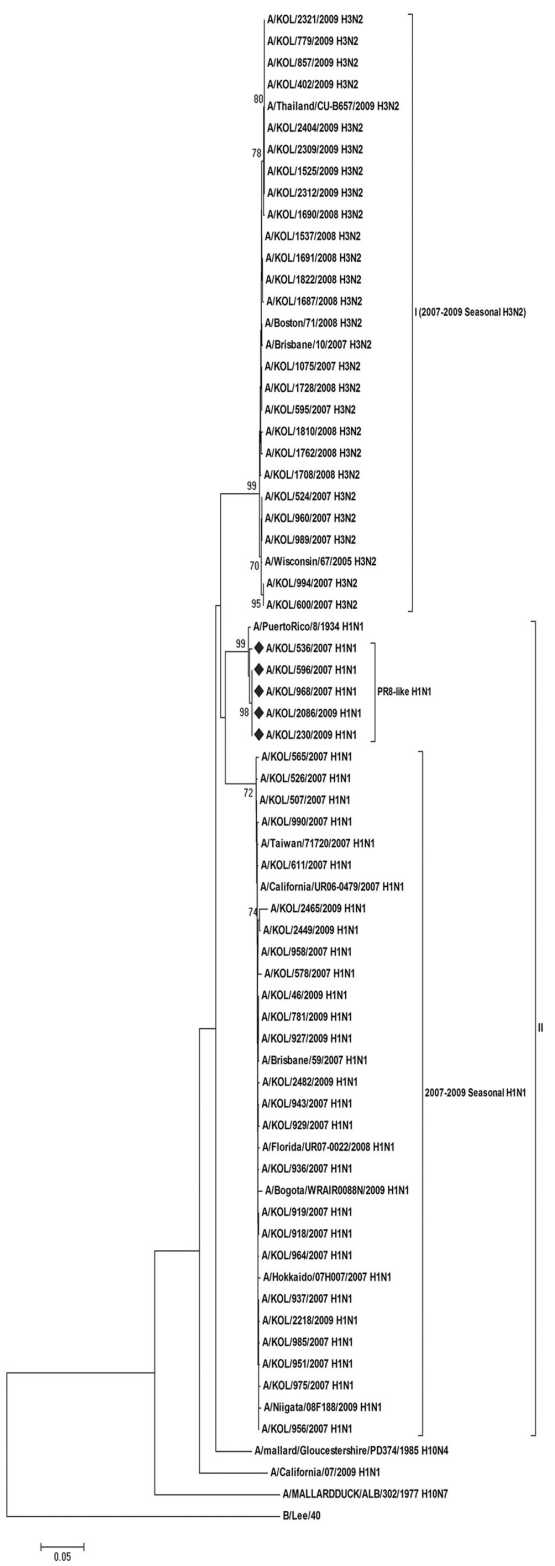
**Phylogenetic analysis of the NS gene**. Phylogenetic analysis of the NS gene (nucleotides) of seasonal influenza A (H1N1/H3N2) strains circulating in eastern India with respect to allele A and allele B gene pool [A/mallard/Gloucestershire/PD374/1985(H10N4) and A/Mallard Duck/ALB/302/1977(H10N7)], concurrent vaccine and other worldwide strains during 2007-2009 was carried out taking B/Lee/40 as an out-group. The tree was created by using neighbor-joining method and bootstrapped values of ≥70% were given for each node. The 2007 and 2009 A/H1N1 eastern India strains with A/Puerto Rico/8/1934(H1N1)-like NS gene are highlighted (♦).

**Figure 2:**
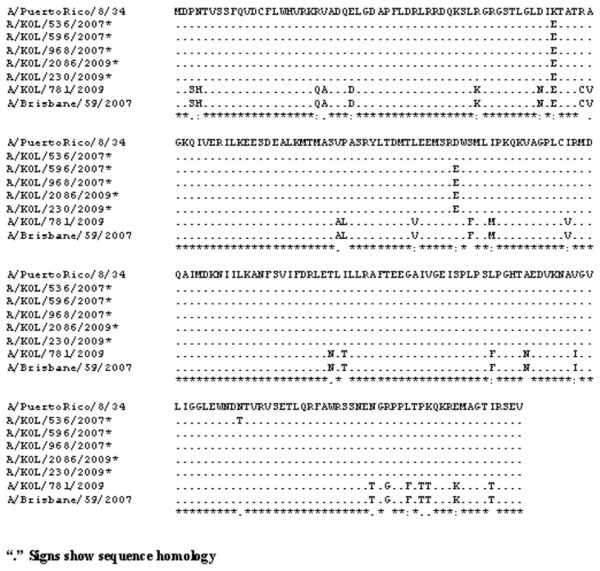
**Alignment of deduced amino acid sequences of NS1 protein**. Alignment of deduced amino acid sequences of NS1 protein of 2009 seasonal Eastern India A/H1N1 strains with respect to the prototype [A/Puerto Rico/8/34(H1N1)] and concurrent vaccine strain [A/Brisbane/59/2007(H1N1)] was carried out. The five 2007 and 2009 seasonal H1N1 Eastern India strains having PR8-like NS1 protein are indicated (*).

Phylogenetic analysis of NS gene sequences comparing different subtypes of influenza A, with respect to B/Lee/40 as an out-group strain, revealed distinct groups within the H1N1 and H3N2 strains of the analyzed eastern India strains (Figure 1). All 24 A/KOL/H3N2 strains analyzed in the study clustered together with A/Wisconsin/67/2005(H3N2) and A/Brisbane/10/2007(H3N2). NS gene of all the A/KOL/H3N2 strains was highly conserved (>97% nucleotide homology). In spite of having evolutionary relationship with the representative strain of NS1 allele A gene pool [21-23], of 31 A/KOL/H1N1 strains, twenty-six strains clustered with 2007-2008 vaccine strains in sub-group 2 of group II, whereas, five strains clustered with A/Puerto Rico/8/34(H1N1) strain in sub-group 1 of group II. These five strains carried NS gene which was similar to PR8-like H1N1 strains, indicating two types of A/H1N1 strains circulating simultaneously. With old strain A/Puerto Rico/8/34(H1N1) as baseline, 25 nucleotide changes were shared among the NS genes of nine H1N1 strains forming a distant branch point of the evolutionary tree. In contrast, nucleotide sequences of NS gene of A/KOL/536/2007(H1N1), A/KOL/596/2007(H1N1), A/KOL/968/2007(H1N1), A/KOL/230/2009(H1N1) and A/KOL/2086/2009(H1N1) were nearly identical with A/Puerto Rico/8/34, differing at only few nucleotide positions (Figure 2 and Table 1). It should be noted that although NS1 gene of these eastern India strains were evolutionarily closer to A/PR/8/34, they were isolated almost 73-75 years later than the prototype. However, HA, NA and M1 gene segments of these five strains were homologous to concurrent A/KOL/H1N1 strains (data not shown).

**Table 1:** Mutations observed in the NS genes of PR8-like A/H1N1 eastern India strains.

Nucleotide mutations	Corresponding amino acid mutations	Name of the strains
C150T	Silent	A/KOL/536/2007(H1N1)
		A/KOL/596/2007(H1N1)
		A/KOL/968/2007(H1N1)

T273C	Silent	
	
C303A	D101E	A/KOL/596/2007(H1N1)
		A/KOL/968/2007(H1N1)
	
G378A	Silent	

A569C	N190T	
		A/KOL/536/2007(H1N1)
	
A594T	Silent	

An alternative method of analysis of the sequence data involves comparison of the silent mutations in the gene sequence since these are not subjected to selective pressure and thus are predicted to be a more reliable marker for evolutionary analysis. Except A/PR/8/34-like eastern India strains, twenty seven silent base changes occurred during the evolution of the NS gene from A/PR/8/34 to A/2007 and/or A/2009 viruses. In essence, comparison of the silent mutations in NS gene sequence of all strains revealed similar evolutionary pattern as compared to one obtained when total nucleotide changes are used.

**Figure 3:**
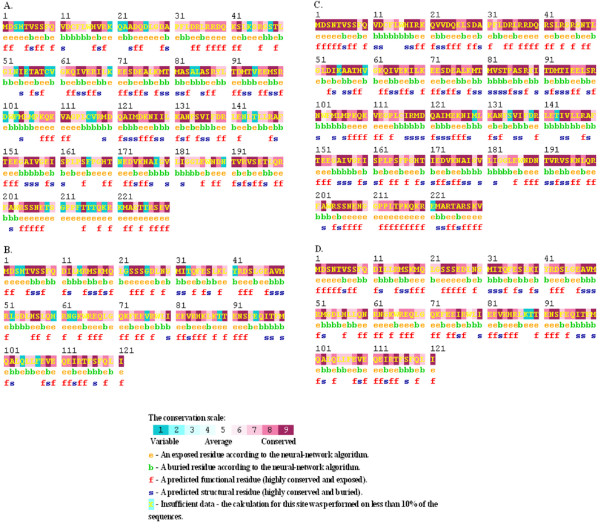
**Result of ConSurf server**. ConSurf server identified the conserved regions among the deduced amino acid sequences of NS1 and NS2 protein of eastern India strains, with respect to concurrent vaccine strains [A/Brisbane/59/2007(H1N1) and A/Brisbane/10/2007(H3N2)]. The degree of conservation was subdivided into nine grades, with grade 1 being the least and grade 9 being most conserved. Figure 3A, 3B and 3C, 3D represents the NS1 and NS2 protein of A/H1N1 and A/H3N2, respectively. Although amino acid residues especially at positions 4, 21, 22, 44, 53, 59, 60, 103 and 106 were conserved among the vaccine strains as well as all other eastern India strains, ConSurf server predicted the lowest score for these amino acid residues suggesting them as highly variable residues of grade 1.

**Figure 4:**
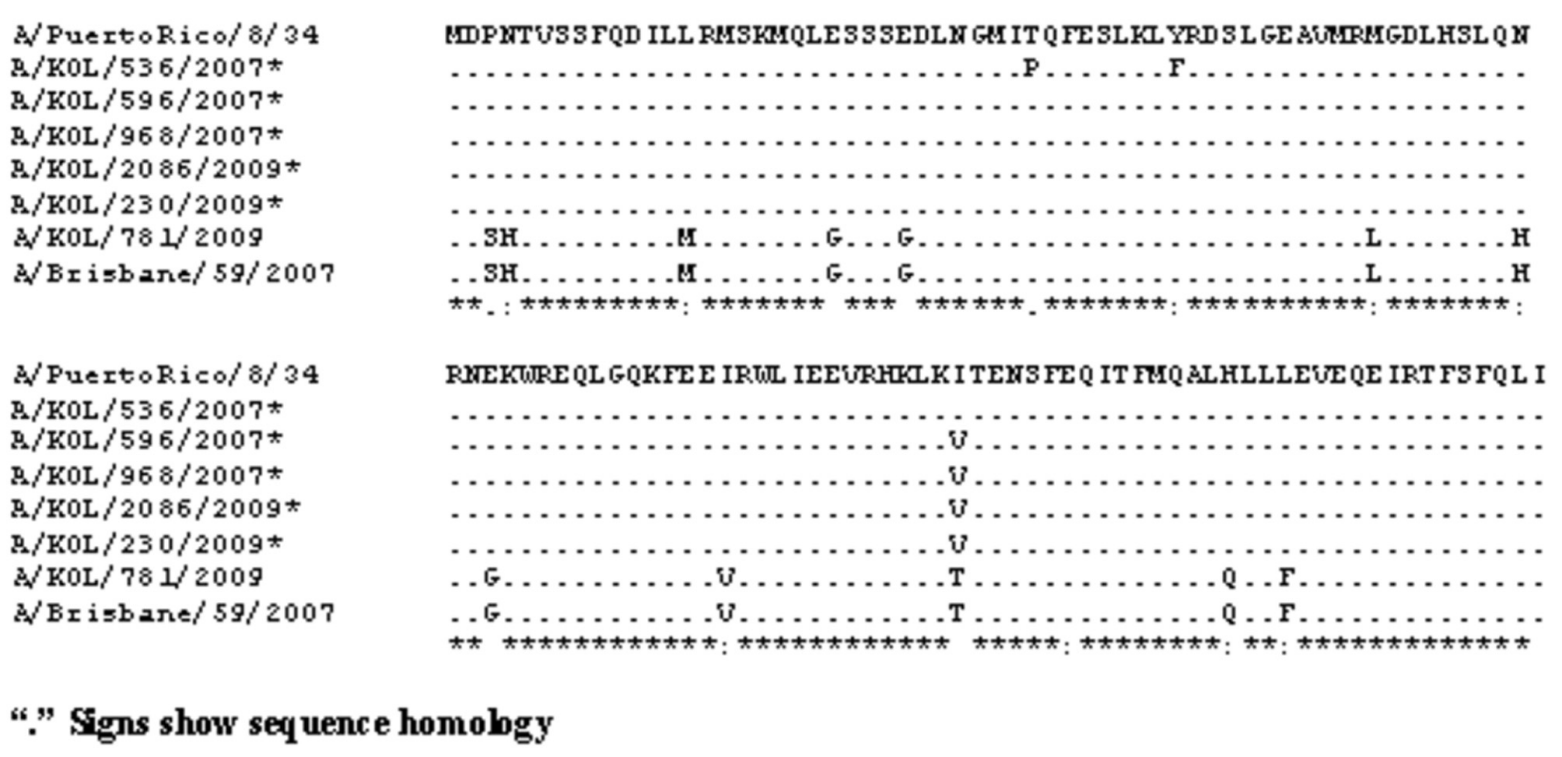
**Alignment of deduced amino acid sequences of NEP/NS2 protein**. Alignment of deduced amino acid sequences of NEP/NS2 protein of 2009 A/H1N1/2009 strains with respect to the prototype [A/Puerto Rico/8/34(H1N1)] and concurrent vaccine strain [A/Brisbane/59/2007(H1N1)] was carried out. The five 2007 and 2009 seasonal H1N1 eastern India strains having PR8-like NEP/NS2 protein are indicated (*).

Figure 3 showed the ConSurf prediction results for the deduced amino acid (aa) sequences of the NS1 and NS2 proteins of A/H1N1 and A/H3N2 with respect to the concurrent vaccine strains. The NS1 polypeptides of three 2007 and two 2009 eastern India H1N1 strains showed sequence homology with the prototype H1N1 strain [A/PR/8/34(H1N1)] with A/PR/8/34(H1N1)-like mutations in both RNA-binding (H4N, Q21R, A22V, K44R, N53D, C59R and V60A) and effector (F103S and M106I) domains, whereas, other strains from this region were more similar to the concurrent vaccine strains (Figure 2). Thus phylogenetic analysis based on amino acid or nucleotide sequences revealed similar evolutionary pattern. In addition, Trp (W) at position 78 of 5 NEP/NS2 which is implicated in NEP-M1 interaction and nuclear export of viral ribonucleoprotein complexes [24] was conserved in all the eastern Indian strains but these five strains had an additional PR8-like G63E substitution in the NEP/NS2 region (Figure 4). Therefore, it can be speculated that these five eastern India strains contain primitive NS gene segment, either due to revert mutations or these strains did not mutate unlike other co-circulating strains.

### Sequence analysis of PB1-F2 gene

**Figure 5:**
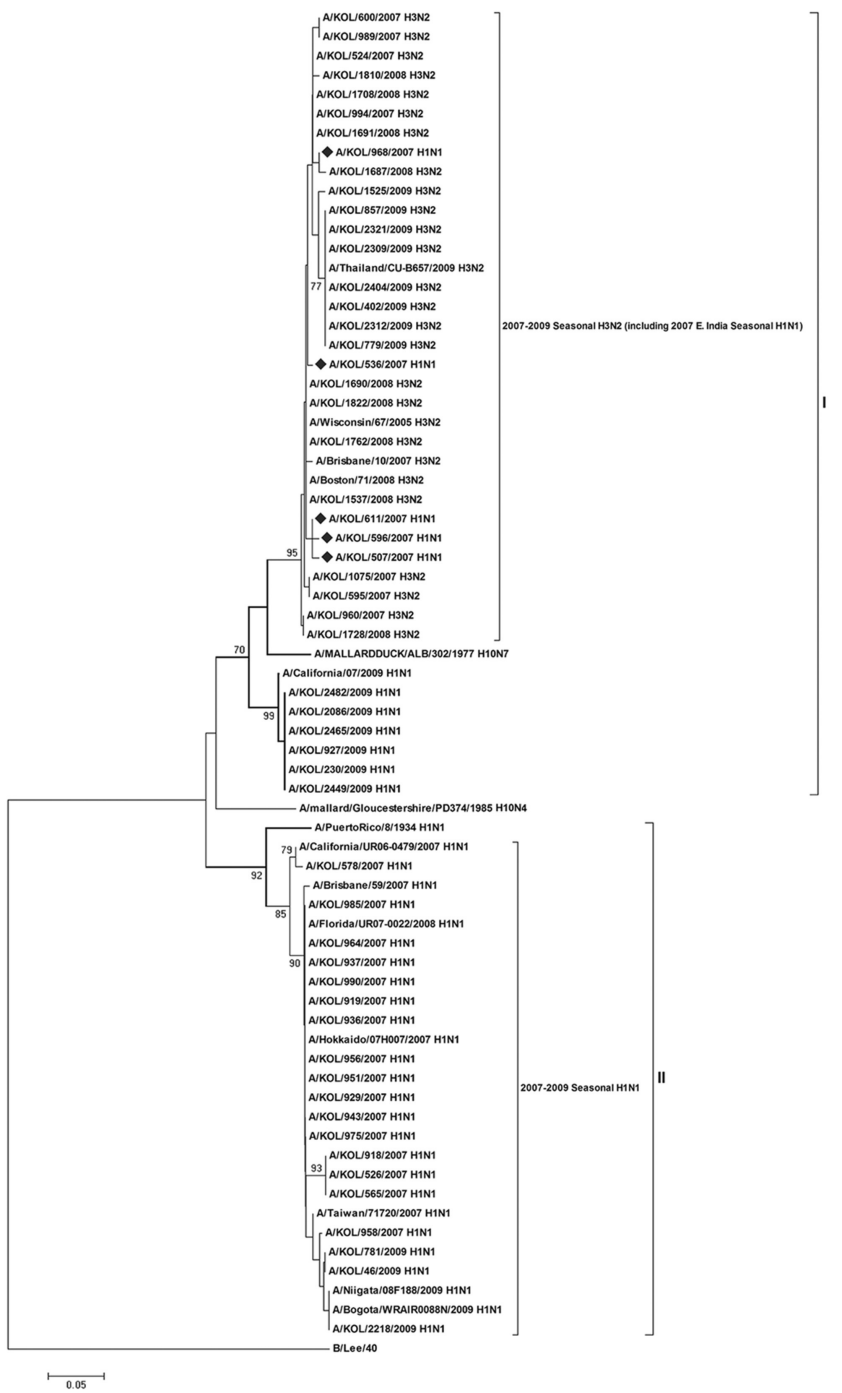
**Phylogenetic analysis of PB1-F2 gene**. Phylogenetic analysis of PB1-F2 gene (nucleotide) of seasonal inf A (H1N1/H3N2) strains circulating in eastern India with respect to representative vaccine, prototype, pH1N1/2009 and concurrent A/H1N1 strains reported worldwide was carried out taking B/Lee/40 as an out-group. The tree was created by using neighbor-joining method and bootstrapped values of ≥70% were given for each node. The 2007 A/H1N1 eastern India strains which showed A/H3N2-like PB1-F2 are highlighted (♦).

Analysis of PB1-F2 gene of A/H1N1 and A/H3N2, considering B/Lee/40 as the out-group strain, revealed two main groups (Figure 5). Group I was represented by the globally reported H3N2 strains including all (n = 24) eastern India strains and representative 2009 pH1N1 strains, whereas, 20/31 A/H1N1 strains of eastern India and other reference A/H1N1 strains clustered in group II. Among rest 11 A/H1N1 strains, 5 strains [A/KOL/507/2007(H1N1), A/KOL/536/2007(H1N1), A/KOL/596/2007(H1N1), A/KOL/611/2007(H1N1) and A/KOL/968/2007(H1N1)] clustered with H3N2 strains in group I, whereas, 6 strains clustered with pH1N1/2009 strains in sub-group 2 within group I as reported earlier [25]. Nucleotide sequence alignment for PB1-F2 gene of the group I H1N1 strains (n = 5) showed only 83%-86% identity with A/Puerto Rico/8/34(H1N1) and the concurrent vaccine strain A/Brisbane/59/2007 (H1N1), compared to 91-94% sequence similarity with co-circulating seasonal A/H3N2 strains, suggesting possible H3N2 origin of PB1-F2 gene segment of these strains.

**Figure 6:**
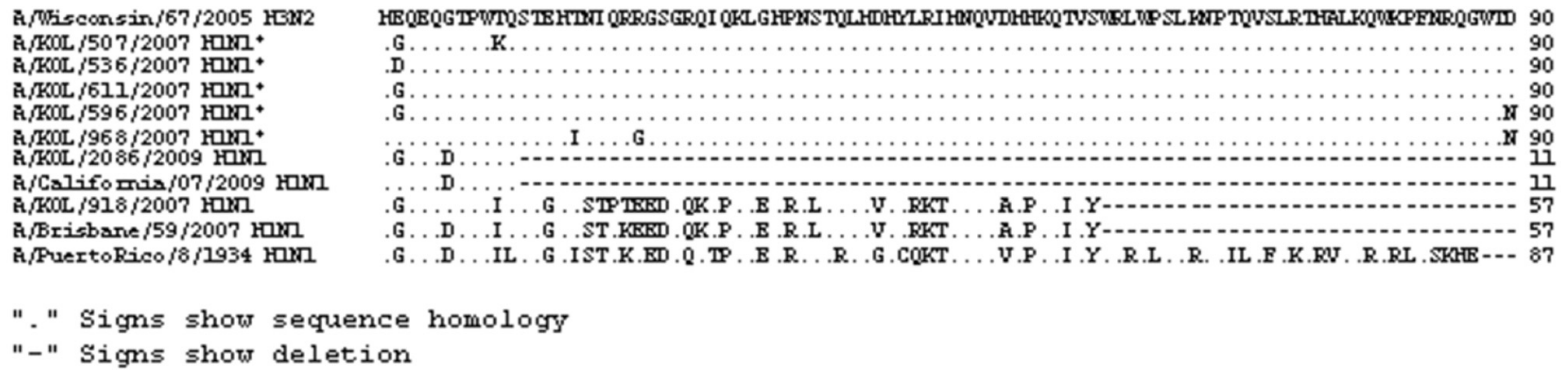
**Alignment of deduced amino acid sequences of PB1-F2 protein**. Alignment of deduced amino acid sequences of PB1-F2 protein of 2007 seasonal Eastern India A/H1N1 strains with respect to the prototype [A/Puerto Rico/8/34(H1N1)] and concurrent vaccine strains [A/Brisbane/59/2007(H1N1); A/Wisconsin/67/2005(H3N2)] was carried out. The five 2007 seasonal H1N1 eastern India strains having A/H3N2-like PB1-F2 protein are indicated (*).

**Figure 7:**
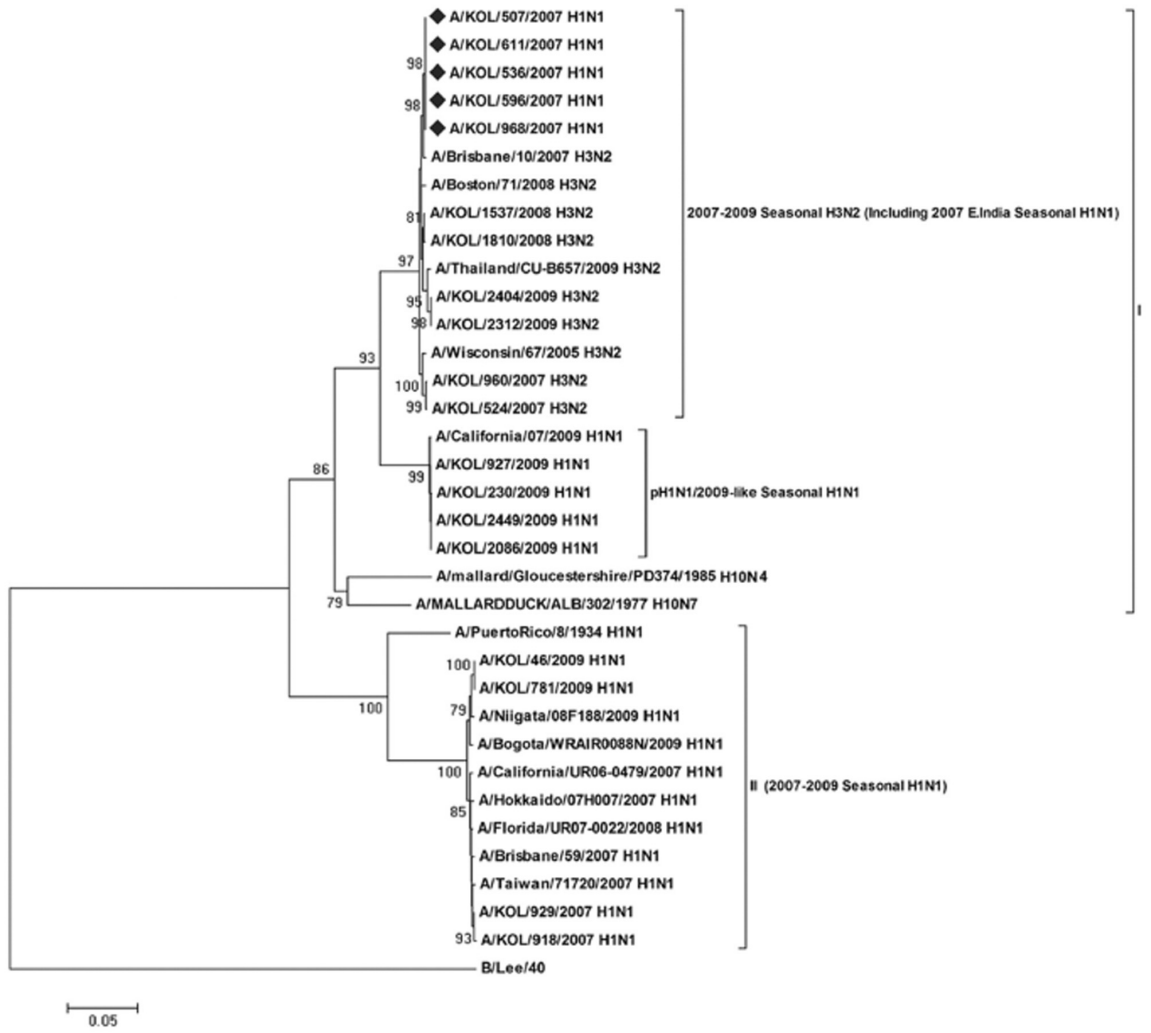
**Phylogenetic analysis of PB1 gene**. Phylogenetic analysis of PB1 gene (nucleotide) of seasonal inf A (H1N1/H3N2) strains circulating in eastern India with respect to representative vaccine, prototype, pH1N1/2009 and concurrent A/H1N1 strains reported worldwide was carried out taking B/Lee/40 as an out-group. The tree was created by using neighbor-joining method and bootstrapped values of ≥70% were given for each node. The 2007 A/H1N1 eastern India strains which showed A/H3N2-like PB1are highlighted (♦).

In majority of strains, the start codon for the PB1 gene is located at nucleotide 25 to 27 and stop codon is at nucleotide 2296 to 2298, resulting in 757 amino acid PB1 protein [26,27]. The complete ORF (119-382) of PB1-F2 in A/Puerto Rico/8/1934 (H1N1) is an 87-residue protein [1]. Deduced amino acids of putative PB1-F2 gene of eastern India strains was compared with respect to A/Wisconsin/67/2005(H3N2). All 31 A/KOL/H1N1 showed an alternative start codon (AUG) at positions 119 to 121 in the PB1 gene, which translated into Met (M) and marked the beginning 6 of PB1-F2 ORF. Consistent with the concurrent vaccine H1N1 strain (A/Brisbane/59/2007(H1N1)), 20/31 H1N1 strains encountered a stop codon (UAG) at positions 290 to 292 resulting in the production of a 57 residue truncated PB1-F2 protein and six A/H1N1 strains from 2009 revealed 11 aa truncated PB1-F2 protein similar to 2009 pandemic H1N1 [25]. Rest five strains [A/KOL/507/2007(H1N1), A/KOL/536/2007(H1N1), A/KOL/596/2007(H1N1), A/KOL/611/2007(H1N1) and A/KOL/968/2007(H1N1)] covered the entire ORF as in these strains, stop codon was observed at aa 391, indicating putative 90 aa PB1-F2 protein, which is three residues longer than PB1-F2 of PR8, but similar to A/Wisconsin/67/2005(H3N2) strain (Figure 6). For confirmation, full length PB1 segment of randomly chosen H1N1 and H3N2 strains including five strains showing H3N2-like PB1-F2 was sequenced. Similar to PB1-F2 results, the full length PB1 gene of these five H1N1 strains clustered with PB1 of H3N2 strains (Figure 7). The multiple alignment result of full-length PB1 sequences of five H3N2-like H1N1 strains confirmed their identity with A/Wisconsin/67/2005(H3N2) rather than that of the concurrent H1N1 vaccine strains suggesting the reassortment event involving PB1 gene between co-circulating H1N1 and H3N2 strains. All the H3N2 strains (n = 24) analyzed in this study, showed full length PB1-F2 ORF (90 aa) which was similar to the concurrent H3N2 vaccine strains.

## Discussion

The complete nucleotide sequence of the NS gene and partial sequence of PB1 gene segment encoding full-length PB1-F2 of representative influenza A (H1N1/H3N2) positive samples collected from the out-patient departments (OPDs) of local hospitals were compared with the concurrent influenza A (H1N1/H3N2) strains, circulating worldwide. Cumulative point mutations and reassortment events due to segmented RNA genome contribute to continuous genetic and antigenic variation in circulating influenza viruses resulting in seasonal epidemics. Unlike HA and NA surface glycoproteins, mutations in the NS genes appeared to be sequential, suggesting that reassortment has probably not contributed significantly to the evolution of the NS gene of these human viruses which is in agreement with previous studies [28,29]. In addition, due to relatively conserved nature of NS gene, reassortment events may have precluded detection. In 1978 recombinant H1N1 viruses with P1, P2, P3 and NP genes from H3N2 still carried HA, NA, M, and NS gene from parent H1N1 subtype [30]. However, significant differences in the NS genes of the influenza A H1N1 and H3N2 subtypes during this study were identified, which allowed detection of an NS gene reassortment [30].

With respect to PB1-F2 protein coding region, five out of thirty-one H1N1 strains (2007-2009) with functional PB1-F2 were evolutionarily close to co-circulating A/H3N2 strains, whereas, corresponding NS gene showed H1N1 origin (Figure 1 and 5). Presumably these five 2007 H1N1 strains arose by reassortment between co-circulating H1N1 and H3N2 viruses in the region. For confirmation HA and NA genes (partial) and M1 (full length) were sequenced, which on analysis confirmed nucleotide identity with A/H1N1 strains. The PB1 gene segment of these strains, however, clustered with A/H3N2 strains suggesting that although these viruses were of H1N1 origin, they probably had derived PB1 segment from an H3N2 virus. The significance of selectively lateral transmission of PB1-F2 gene among co-circulating strains is not clear, but since PB1-F2 protein is associated with pathogenesis, it may confer improved infectivity or replication efficiency. As reported earlier by our group [25], six A/H1N1 strains had truncated 11 aa PB1-F2 similar to 2009 pH1N1 viruses. Rest twenty A/H1N1 strains with 57 aa PB1-F2 peptide were similar to the concurrent A/H1N1 vaccine strains (Figure 6).

Surprisingly, NS nucleotide sequences of five A/H1N1 strains was highly homologous (>97%) with the 1934 prototype strain [A/Puerto Rico/8/34(H1N1)] (Figure 2). In addition, these five strains contained G63E substitution in NEP, similar to the highly pathogenic avian influenza H5N1 viruses, which may confer higher pathogenicity [31]. To verify possible cross contamination, BLAST search of HA, NA, M1 and NS1 gene sequences showed only NS1 having sole identity with A/PR/8/34(H1N1). Thus, a chance of cross contamination with laboratory PR8 strain was ruled out. Though the frequency of vaccination in India is very low but since the WHO approved vaccines with PR8 backbone are used, possibility of reassortment with the vaccine strain can not be ruled out. Moreover, 3 out of five PR8-like NS1 carrying A/H1N1 2007 strains, had H3N2-like PB1-F2 gene, whereas, 2/5 had non-functional PB1-F2 similar to pandemic A/H1N1 strains of 2009 (Figure 1 and 5). Thus the circulation of prototype NS gene carrying A/H1N1 strains in 2007 and 2009, with PB1-F2 gene from diverse origin underlines the complexity of influenza virus genetics and evolution. In contrast to A/H1N1 strains, all A/H3N2 (n = 24) strains analyzed in this study revealed highly conserved NS and PB1-F2 gene, with >98.5% homology to concurrent A/H3N2 strains circulating worldwide.

## Conclusion

Thus, it can be hypothesized that NS and PB1-F2 gene segments of H3N2 viruses are evolutionarily more stable. This is in contrast to the analysis of HA and NA genes in the region, where comparative amino acid mutation rates were observed in both H1N1 and H3N2 strains [32]. Reassortment events not involving the surface glycoproteins HA and NA largely remain undetected due to specific use of HA and NA specific antisera or sequencing primers for identification of circulating strains in most countries. This study highlights the existence of A/H1N1 and A/H3N2 viruses with viral virulence marker genes PB1 and NS from diverse origin co-circulating in the same geographical location. Therefore, analysis of gene segments other than HA and NA genes, is important to understand evolution of strains with variable pathogenic potential.

## Methods

### Ethics Statement

The study was approved by the Institutional Ethics Committee, National Institute of Cholera & Enteric Diseases (Indian Council of Medical Research). The concerned members of this committee are: Hon'ble Justice Pinaki Ghosh, Kolkata High Court (Chairman), Dr. D. Mahalanabis (Member), Prof. A. C. Ghosh (Member), Prof. Biswapati Mukherjee (Member), Prof. Mrinmoy Ghosh (Member), Prof. Mrinal Kanti Chatterjee (Member), Mr. M. A. Wohab (Member), Mr. Amitrajit Ukil (Member), Mrs. Debolina Sarkar (Member), Prof. Subir Kumar Dutta (Member) and Dr. Phalguni Dutta (Member Secretary). Written informed consent was obtained from all participants involved in this study.

### Sample collection

Nasal and throat swabs were collected in Viral Transport Medium (VTM) from patients with influenza-like illness reporting in outpatient's ward of two referral hospitals; Dr. B.C. Roy Memorial Hospital for children (BCRMHC) and R.G. Kar Medical College and Hospital (RGKMCH) during 2007- 2009, as described previously [32]. Of 166 influenza A positive samples, 55 samples were picked randomly (31 H1N1 and 24 H3N2) for sequencing of PB1-F2 and NS genes. For confirmation, total PB1 gene segment was sequenced in 19 strains chosen randomly. All sequences were submitted to Genbank and compared for nucleotide and amino acid homology.

### Viral RNA Extraction

Extraction of viral RNA from the clinical samples was carried out using commercially available QiaAmp Viral RNA Mini Kit (Qiagen, GmbH, Hilden, Germany) according to the manufacturer's instruction.

### Amplification of virus genes

To study the genetic diversity, full length NS, PB1, M1 and PB1-F2 encoding gene segments were amplified by RT-PCR using RevertAid™ First Strand cDNA Synthesis kit and DreamTaq™ DNA Polymerase (Fermentas Life Sciences, Burlington, Canada) as per kit protocol with specific primers for the mentioned gene segments [33,34]. PCR products were purified by column purification using QIAquick PCR Purification Kit (Qiagen, GmbH, Hilden, Germany).

### Sequencing and phylogenetic analysis

Genes were sequenced following dideoxynucleotides chain termination method of Sanger *et al*. (1977), in ABI Prism automated 3100 DNA sequencer (Applied Biosystem, Foster City, USA) using Big-Dye Terminator Chemistry. Sequences were compared with published cognate sequences of corresponding genes. DDBJ (DNA Data Bank of Japan) Clustal W system (version 1.83) was used for multiple sequence alignment of different nucleotide sequences of eastern India strains with other reference strains. Neighbour-Joining (N-J) trees were generated using pair-wise gap deletion, Maximum Composite Likelihood as distance measure and 1000 boot-strap replicates (generated with MEGA4) with boot strap values ≥70%.

### Identification of Conserved Regions

To study the conservation among the NS1 and NS2 sequences of eastern India strains, amino acid (aa) sequences of eastern India strains were deduced by the DNA sequence translation tool EMBOSS-Transeq (EBI Group). Conserved regions were identified and mapped onto the protein structures using the web-based ConSurf server (http://consurf.tau.ac.il/) [35,36] providing the multiple sequence alignment as input. The degree of conservation was subdivided into nine grades, with grade 1 being the least and grade 9 being the most conserved.

### Accession Numbers for nucleotide sequences

Accession numbers of all newly reported sequences obtained for full length non-structural (NS) gene, full length polymerase basic protein 1 (PB1) gene, PB1-F2 gene and matrix (M1) gene in this study are: [GenBank: HQ853495 to HQ853549; HM460507, HM460510, HQ154076, HQ154078, HQ154084, HQ853550, HQ853552, HQ853555, HQ853556, HQ853557, HQ853559, HQ853567, HQ853571, HQ853573, HQ853574, HQ853579, HQ853586, HQ853589, HQ853591; HQ853551, HQ853553, HQ853554, HQ853558, HQ853560 to HQ853566, HQ853568 to HQ853570, HQ853572, HQ853575 to HQ853578, HQ853580 to HQ853585, HQ853587, HQ853588, HQ853590, HQ853592 to HQ853595, HM460508, HM460509, HM460511, HQ154075; JF903566 to JF903570].

## Competing interests

The authors declare that they have no competing interests.

## Authors' contributions

MS and MCS conceived and designed experiments. JM and AG were involved in screening patients in hospitals and collecting clinical data. Experiments were executed by MS and SC. Analysis of results and drafting manuscript was done by MCS and MSC. SC, ACM and MCS contributed towards inputs in data interpretation, reviewing final manuscript and overall administrative responsibilities of the project. All authors read and approved the final manuscript.
